# Association between four common microRNA polymorphisms and the risk of hepatocellular carcinoma and HBV infection

**DOI:** 10.3892/ol.2014.2257

**Published:** 2014-06-16

**Authors:** JIAN-TAO KOU, HUA FAN, DONGDONG HAN, LIXIN LI, PING LI, JIQIAO ZHU, JUN MA, ZHI-HUA ZHANG, QIANG HE

**Affiliations:** Department of Hepatobiliary Surgery, Beijing Chaoyang Hospital, Capital Medical University, Beijing 100020, P.R. China

**Keywords:** microRNA, polymorphisms, hepatocellular carcinoma, hepatitis B virus

## Abstract

microRNAs (miR/miRNAs) have been demonstrated to function as tumor suppressors and oncogenes, and miRNA polymorphisms may have a role in cancer development. The present study aimed to investigate the association between the miR-146aG>C, miR-149C>T, miR-196a2C>T and miR-499A>G polymorphisms and the risk of hepatocellular carcinoma (HCC) and hepatitis B virus (HBV) infection. A total of 271 patients with HCC and 532 healthy control participants were enrolled in the present study. miR-146aG>C, miR-149C>T, miR-196a2C>T and miR-499A>G polymorphisms were genotyped using the polymerase chain reaction-restriction fragment length polymorphism method. A significant difference was identified in the genotype frequency of miR-196a2C>T in the patients in the case group compared with the control group (χ^2^=6.88; P=0.032). Compared with the CC genotype, the miR-196a2 TT genotype was associated with a significantly reduced risk of HCC [odds ratio (OR), 0.62; 95% confidence interval (CI), 0.38–0.99], and a significantly reduced risk was also found in the dominant (OR, 0.69; 95% CI, 0.49–0.98) and recessive (OR, 0.70; 95% CI, 0.46–1.02) models. Moreover, individuals with HBV who were carrying the miR-196a2 CT and TT genotypes had a significantly reduced risk of HCC (OR, 0.62; 95% CI, 0.41–0.95; and OR, 0.39; 95% CI, 0.20–0.73, respectively). In conclusion, the present study found that the miR-196a2C>T polymorphism has a protective effect in patients with HCC, particularly in those with HBV infection.

## Introduction

Hepatocellular carcinoma (HCC) is the fifth most common type of malignant tumor worldwide and has the second highest mortality rate, with an estimated 293,318 new cases in China per year (1). The primary risk factors for HCC are chronic hepatitis B virus (HBV) and hepatitis C virus (HCV) infection; however, only ~10% of patients infected with HBV and HCV develop HCC during their lifetime (2,3). Thus, certain genetic and environmental factors may also be involved in the development of HCC (2).

microRNAs (miR/miRNAs) are a novel class of endogenous, non-coding RNAs that regulate gene expression at the post-transcriptional level through repressing translation or decreasing mRNA stability (4,5). miRNAs have been demonstrated to play a role in several processes, including development, apoptosis, proliferation and differentiation, in the eukaryotic cells of various organisms (6,7). A number of previous studies have analyzed the association between miRNAs and the susceptibility and prognosis of various types of human cancer (8–10). The miR-146aG>C, miR-149C>T, miR-196a2C>T and miR-499A>G polymorphisms have been reported to be associated with various types of cancer, including lung, breast, colorectal and gastric cancer (11–14). Several more recent studies have reported that miRNA polymorphisms are associated with the risk of HCC; however, the results are inconsistent (15–18). Moreover, several studies have demonstrated that miRNAs act as repressors in viral infection pathways, and that viruses have miRNAs which regulate gene expression, thus miRNAs contribute to the pathogenicity of the virus (20,21). miRNAs may therefore be key regulators in host-virus interactions and in the regulation of viral replication. In the present study, it was hypothesized that polymorphisms in miR-146aG>C, miR-149C>T, miR-196a2C>T and miR-499A>G may have an effect on the risk of HCC and the interaction with HBV infection. In order to investigate this hypothesis, a case-control study was performed to investigate the association between four common miRNA polymorphisms and the risk of HCC.

## Materials and methods

### Study population

A total of 302 individuals were periodically enrolled in the present study between January 2010 and February 2012. HCC diagnoses were based on liver biopsies or at least two radiological tests for HCC, including abdominal ultrasound, spiral computed tomography, magnetic resonance imaging and hepatic angiography, or by increased α-fetoprotein levels (≥200 μg/ml). The control group consisted of 568 individuals randomly selected from the health examination center at Beijing Chaoyang Hospital of Capital Medical University (Beijing, China). None of the control subjects had a history of cancer, liver disease, kidney disease, coronary artery disease or other metabolic disorders.

Serum hepatitis B surface antigen and anti-HCV antibody levels were assessed with a microparticle enzyme immunoassay using commercial assay kits to determine the infection of HBV or HCV. The clinical characteristics of the patients with HCC were obtained using medical records. The demographic characteristics were collected using a self-designed questionnaire, which included questions on smoking status and alcohol consumption. The present study was approved by the Medical Ethical Committee of Beijing Chaoyang Hospital of Capital Medical University, and written informed consent with regard to the use of patient blood samples for research studies was obtained from all participants.

### DNA extraction and genotyping

All participants provided 5 ml venous blood, and blood samples were stored at −20°C with 0.5 mg/ml EDTA as an anticoagulant, until required. Genomic DNA was extracted using the TIANamp Blood DNA kit (Tiangen Biotech Co., Ltd., Beijing, China), according to the manufacturer’s instructions. Duplex polymerase chain reaction (PCR) with confronting two-pair primers was used for PCR-restriction fragment length polymorphism analysis in order to analyze the miR-146aG>C, miR-149C>T, miR-196a2C>T and miR-499A>G genotypes. The primers used and the products generated for the amplification of miR-146aG>C, miR-149C>T, miR-196a2C>T and miR-499A>G are shown in Table I.

The following PCR cycling conditions were used: An initial melting step of 5 min at 95°C, followed by 35 cycles of denaturation at 94°C for 30 sec and annealing at 64°C for 30 sec, with a final extension at 72°C for 10 min. Reproducibility was verified using repeat analysis of a randomly selected subgroup of 10% of the subjects.

### Statistical analysis

Data analysis was performed using SPSS version 10.0 (SPSS, Inc., Chicago, IL, USA) for Windows. Continuous variables are presented as the mean ± standard deviation, and categorical variables are presented as frequencies and percentages. Differences in the distribution of demographic characteristics between the case and control groups were assessed using χ^2^ tests for the categorical data and Student’s t-tests for the continuous variables. The χ^2^ test was used to compare the Hardy-Weinberg equilibrium of the genotype frequencies of miR-146aG>C, miR-149C>T, miR-196a2C>T and miR-499A>G in the control group. The association between miR-146aG>C, miR-149C>T, miR-196a2C>T or miR-499A>G polymorphisms and the risk of HCC was estimated using odds ratios (ORs) and their 95% confidence intervals (CIs) from conditional logistic regression analyses. A homozygous genotype was used as the reference for calculating ORs. All P-values were two sided and P<0.05 was considered to indicate a statistically significant difference.

## Results

Among the 302 patients with HCC who were screened, 271 were included in the present study, with a participation rate of 89.7%. For the control group, 568 individuals were screened and 532 were recruited into the present study, with a participation rate of 93.7%. The HCC group consisted of 72 females and 199 males, while the control group consisted of 206 females and 326 males (Table II). The mean ages in the HCC and control groups were 55.8±10.6 and 52.6±11.2 years, respectively. The patients with HCC were more likely to be male, with a higher age and incidence of HBV and HCV infection and a high probability of having a family history of cancer (All P<0.05). No significant difference was observed in the smoking and drinking statuses of the individuals in the case group compared with those in the control group (All P>0.05). For the clinical characteristics, 204 patients (75.3%) displayed liver cirrhosis, 151 (55.7%) were TNM stage III–IV, 141 (52.0%) were classified as Child-Pugh class C, 91 (33.6%) were classified as Child-Pugh class B, 116 (42.8%) had α-Fetoprotein levels of <100 ng/ml and 107 (39.5%) had levels of >400 ng/ml.

The allele and genotype distributions of miR-146aG>C, miR-149C>T, miR-196a2C>T and miR-499A>G were found to be in Hardy-Weinberg equilibrium in the control group (Table III). The miR-196a2C>T genotype frequency was significantly different in the individuals in the case group compared with those in the control group (χ^2^=6.88; P=0.032), while the frequencies of miR-146aG>C, miR-149C>T and miR-499A>G showed no significant differences between the case and control groups. Multivariate regression analyses revealed that subjects carrying the miR-196a2 TT genotype had a significantly reduced risk of HCC, with an adjusted OR (95% CI) of 0.62 (0.38–0.99), and a significantly reduced risk was found in the dominant (OR, 0.69; 95% CI, 0.49–0.98) and recessive (OR, 0.70; 95% CI, 0.46–1.02) models. However, no significant association was found between the miR-146aG>C, miR-149C>T or miR-499A>G polymorphisms and the risk of HCC.

Further analysis was performed on the interaction between miR-196a2C>T and the HBV and HCV infections (Table IV). Compared with the miR-196a2 CC genotype, individuals with HBV carrying the miR-196a2 CT and TT genotypes had a significantly reduced risk of HCC, with an adjusted OR (95% CI) of 0.62 (0.41–0.95) and 0.39 (0.20–0.73), respectively. Moreover, the miR-196a2 T allele was found to significantly reduce the risk of HCC by 0.33-fold, compared with the C allele. However, no significant association was found between miR-196a2C>T and HCC risk in patients with the HCV infection or those without HBV and HCV infection.

## Discussion

In the present case-control study, the miR-196a2C>T polymorphism was found to be associated with a significantly reduced risk of HCC, however, miR-146aG>C, miR-149C>T and miR-499A>G were not associated with this risk. Furthermore, the miR-196a2C>T polymorphism was observed to significantly reduce the risk of HCC in patients infected with HBV.

It is well known that cancer is caused by multiple factors, including environmental and genetic factors. It has also been reported that miRNAs are a group of small non-coding RNA molecules, which have an important role in the development of cancer through regulating the expression of tumor suppressor genes (22). Previous clinical experimental studies have shown that polymorphisms in miR-122, -196, -423 and -499 are associated with the risk of hepatocellular carcinoma (23–25). The reasons for this may be that single nucleotide polymorphism (SNP) miRNA sequences can change the expression and maturation of miRNA, and thus, increase the risk of carcinogenesis (26).

The present study demonstrated that miR-196a2C>T was associated with HCC risk. It is reported that miR 196a2C>T polymorphisms have a role in the development of several types of cancer, including colorectal, breast, pancreastic, gastrointestinal and lung cancer (27–32). For HCC, only three studies have investigated the association between miR 196a2C>T polymorphisms and the development of HCC (15,16, 33). In a study performed in Chinese, HBV-related patients with HCC showed that the risk of HCC was significantly higher in patients with the miR 196a2 CT genotype or T allele compared with those with the CC genotype (15). Furthermore, a study on 310 patients with HCC and 222 controls reported that the miR 196a2 CC genotype was associated with significantly increased mature miR 196a expression and that the miR 196a2 polymorphism may contribute to cirrhosis-related HCC susceptibility through affecting mature miR 196a expression (16). The present study found that the miR 196a2 TT genotype and the T allele significantly reduced the risk of HCC compared with the CC genotype, which is in accordance with certain other previous studies (15,16). However, in another study conducted in Chinese, HCC patients showed that the miR 196a2 TT genotype and the T allele greatly increased the risk of HCC (33). Furthermore, a recent study has shown that the miR 196a2C>T polymorphism may not be a HCC susceptibility factor, but may affect the effects of the HBV mutations (34). Discrepancies of these results may be due to differences in variant frequencies among various ethnic groups, and miR 196a2C>T polymorphisms may have different roles in the development of HCC depending on the population.

In the present study, miR-196a2C>T was found to interact with HBV infection. A previous study has shown that the miR-196a2C>T polymorphism is strongly affected by HBV mutations (34), which suggested that the HBV mutation may act synergistically with the miR-196a2C>T polymorphism.

The present study was performed in a single hospital and the number of patients included was relatively small. The relatively small sample size may decrease the statistical power of the investigation on the role of the miR-146aG>C, miRNA-149C>T and miR-499A>G genetic alterations. In the present study, a number if the patients who were newly diagnosed with HCC left the study. This included patients who moved to other hospitals or did not agree to provide genetic information and blood samples. Thus, further large, multicenter investigations are required to confirm the association between SNPs in miRNA and the risk of HCC.

In conclusion, in the present study, the miR-196a2C>T polymorphism was found to have a protective role in patients with HCC, particularly in those with HBV infection. However, no association was found between the miR-146aG>C, miRNA-149C>T or miR-499A>G polymorphisms and the risk of HCC. SNPs in miRNA sequences may be used as diagnostic biomarkers for HCC. Further large-sample investigations are required to investigate the role of SNPs in miRNA sequences in the development of HCC.

## Figures and Tables

**Table I:** Primer sequences used for miRNA amplification.

Gene variants	Primer sequence (5′-3′)	Product, bp
miR-146aG>C		147
Forward	5′-CAA AGT CTT CACTTC CCT GCC A-3′	
Reverse	5′-GAT GTT TAA CTC CTC TCC ACG TGA TC-3′	
miR-149C>T		263
Forward	5′-CTG GCT CCG TGT CTT CAC TC-3′	
Reverse	5′-TGA GGC CCG AAACAC CCG TA-3′	
miR-196a2C>T		149
Forward	5′-CCC CTT CCC TTC TCC TCC AGA TA-3′	
Reverse	5′-CGA AAA CCG ACT GAT GTA ACT CCG-3′	
miR-499A>G		146
Forward	5′-CAA AGT CTT CAC TTC CCT GCC A-3′	
Reverse	5′-GAT GTT TAA CTC CTC TCC ACG TGA TC-3′	

miR/miRNA, microRNA.

**Table II:** Clinicopathological characteristics in the patients (n=271) with HCC and the control participants (n=532).

Variables	Cases	%	Controls	%	χ^2^ or t-value	P-value
Age, years (mean ± SD)	55.8±10.6	-	52.6±11.2	-	3.89	<0.001
Gender, n						
Male	199	73.4	326	61.3	-	-
Female	72	26.6	206	38.7	11.72	0.001
Smoking, n						
No	173	63.8	354	66.5	-	-
Yes	98	36.2	178	33.5	0.70	0.41
Drinking, n						
No	182	67.2	389	73.1	-	-
Yes	89	32.8	143	26.9	2.07	0.15
Family history of cancer, n						
No	249	91.9	529	99.4	-	-
Yes	22	8.1	3	0.6	37.04	<0.001
Viral infection, n						
Both negative	58	21.4	481	90.4	-	-
HBsAg-positive	159	58.7	43	8.1	-	-
Anti-HCV Ab-positive	49	18.1	8	1.5	-	-
Both positive	5	1.8	0	0.0	389.37	<0.001
Liver cirrhosis, n						
Absent	204	75.3	-	-	-	-
Present	67	24.7	-	-	-	-
TNM stage, n						
I–II	151	55.7	-	-	-	-
III–IV	120	44.3	-	-	-	-
Child-Pugh classification, n						
A	39	14.4	-	-	-	-
B	91	33.6	-	-	-	-
C	141	52.0	-	-	-	-
α-Fetoprotein, ng/ml						
<100	116	42.8	-	-	-	-
100–400	48	17.7	-	-	-	-
>400	107	39.5	-	-	-	-

HCC, hepatocellular carcinoma; SD, standard deviation; HBsAg, hepatitis B surface antigen; Ab, antibody; HCV, hepatitis C virus; TNM, tumor-node-metastasis.

**Table III:** Comparison of the genotype frequencies and ORs of four miRNA polymorphisms in the case group (n=271) compared with the control group (n=532).

							OR (95% CI)^a^		
							
Genotype	Controls, n	%	Cases, n	%	χ^2^	P-value	Codominant	Dominant	Recessive
miR-146aG>C									
CC	179	33.6	99	36.5	-	-	-	-	-
CG	297	55.8	147	54.2	-	-	0.89 (0.64–1.24)	0.88 (0.64–1.21)	0.88 (0.53–1.48)
GG	56	10.5	25	9.2	0.66	0.72	0.82 (0.47–1.44)	-	-
miR-149C>T									
CC	202	38.0	113	41.7	-	-	-	-	-
CT	253	47.6	122	45.0	-	-	0.87 (0.63–1.21)	0.86 (0.63–1.17)	0.87 (0.56–1.37)
TT	77	14.5	35	12.9	1.13	0.57	0.81 (0.50–1.32)	-	-
miR-196a2C>T									
CC	125	23.5	84	31.0	-	-	-	-	
CT	304	57.1	150	55.4	-	-	0.74 (0.52–1.07)	0.69 (0.49–0.98)	0.70 (0.46–1.02)
TT	103	19.4	37	13.7	6.88	0.032	0.55 (0.34–0.89)	-	-
miR-499A>G									
AA	391	73.5	210	77.5	-	-	-	-	-
AG	110	20.7	49	18.1	-	-	0.83 (0.56–1.22)	0.81 (0.57–1.15)	0.75 (0.38–1.55)
GG	31	5.8	12	4.4	1.34	0.51	0.72 (0.36–1.50)	-	-

a Adjusted for gender, age and family history of cancer.

miR/miRNA; microRNA; OR, odds ratio; CI, confidence interval.

**Table IV:** miR-196a2C>T polymorphism and HCC risk stratified by HBV and HCV infection.

Genotype	Control (n=532)	%	HBV− and HCV− negative (n=58)	%	OR (95% CI)^a^	HBV (n=159)	%	OR (95% CI)^a^	HCV (n=49)	%	OR (95% CI)^a^
miR-196a2C>T											
CC	125	23.5	16	27.6	1.00 (Reference)	56	35.2	1.00 (Reference)	16	32.7	1.00 (Reference)
CT	304	57.1	32	55.2	0.82 (0.42–1.67)	85	53.5	0.62 (0.41–0.95)	26	53.1	0.67 (0.33–1.38)
TT	103	19.4	10	17.2	0.76 (0.29–1.87)	18	11.3	0.39 (0.20–0.73)	7	14.3	0.53 (0.18–1.43)
C allele	554	52.1	64	55.2	1.00 (Reference)	197	61.9	1.00 (Reference)	58	59.2	1.00 (Reference)
T allele	510	47.9	52	44.8	0.88 (0.59–1.32)	121	38.1	0.67 (0.51–0.87)	40	40.8	0.75 (0.48–1.16)

a Adjusted for gender, age and family history of cancer.

miRNA; microRNA; OR, odds ratio; CI, confidence interval; HBV, hepatitis B virus; HCV, hepatitis C virus; HCC, hepatocellular carcinoma.
